# Polyisoprenylated cysteinyl amide inhibitors deplete singly polyisoprenylated monomeric G-proteins in lung and breast cancer cell lines

**DOI:** 10.18632/oncotarget.28390

**Published:** 2023-03-24

**Authors:** Nada Tawfeeq, Jassy Mary S. Lazarte, Yonghao Jin, Matthew D. Gregory, Nazarius S. Lamango

**Affiliations:** ^1^Florida A&M University College of Pharmacy Pharmaceutical Sciences, Institute of Public Health, Tallahassee, FL 32307, USA; ^2^Department of Pharmaceutical Chemistry, College of Clinical Pharmacy, Imam Abdulrahman bin Faisal University, Dammam, Eastern Province, Kingdom of Saudi Arabia; ^*^These authors contributed equally to this work and share first authorship

**Keywords:** PCAIs, G-proteins, KRAS, RHOA, RAC1

## Abstract

Finding effective therapies against cancers driven by mutant and/or overexpressed hyperactive G-proteins remains an area of active research. Polyisoprenylated cysteinyl amide inhibitors (PCAIs) are agents that mimic the essential posttranslational modifications of G-proteins. It is hypothesized that PCAIs work as anticancer agents by disrupting polyisoprenylation-dependent functional interactions of the G-Proteins. This study tested this hypothesis by determining the effect of the PCAIs on the levels of RAS and related monomeric G-proteins. Following 48 h exposure, we found significant decreases in the levels of KRAS, RHOA, RAC1, and CDC42 ranging within 20–66% after NSL-YHJ-2-27 (5 μM) treatment in all four cell lines tested, A549, NCI-H1299, MDA-MB-231, and MDA-MB-468. However, no significant difference was observed on the G-protein, RAB5A. Interestingly, 38 and 44% decreases in the levels of the farnesylated and acylated NRAS were observed in the two breast cancer cell lines, MDA-MB-231, and MDA-MB-468, respectively, while HRAS levels showed a 36% decrease only in MDA-MB-468 cells. Moreover, after PCAIs treatment, migration, and invasion of A549 cells were inhibited by 72 and 70%, respectively while the levels of vinculin and fascin dropped by 33 and 43%, respectively. These findings implicate the potential role of PCAIs as anticancer agents through their direct interaction with monomeric G-proteins.

## INTRODUCTION

Small G-proteins, monomeric GTPases, or the RAS (Rat sarcoma) superfamily are a large family of small guanine nucleotide-binding proteins with molecular weights ranging from 20 to 30 kDa [1, 2]. These proteins share a core structure, the conserved G-box (GDP/GTP) binding domain, of approximately 170 residues [3]. Small GTPases function as binary molecular switches, transmitting extracellular signals to an intracellular environment [4]. The superfamily is classified into five subfamilies based on the cellular processes that they regulate [2]. These include the founding member of the superfamily RAS [5], RHO (Ras homology) [6], ARF/SAR (adenosine diphosphate (ADP) ribosylation factor) [7], the largest subfamily RAB (RAS-like in brain or RAS-related in brain) [8] and RAN (RAS-like nuclear or RAS-related nuclear protein) [9]. The small GTPase families regulate a wide range of processes in the cell; however, each family performs different functions within the cell due to differences in their structures, post-translational modifications (PTMs), and subcellular localization (Table 1) [10].

**Table 1:** Cellular functions and mutation and overexpression incidence rates of G-proteins in human cancers

Subfamily	Cellular functions	G-protein	Genetic alteration in cancer	Major cancer type	Incidence rates (%)	References
RAS	Mediate and activate some major pathways that control cell proliferation, survival, and cell cycle progression [4].	KRAS4b	Mutation	Pancreatic cancer	90	[28–31]
				Non-small cell lung cancer	30–35	
		KRAS4a		Colorectal Colon cancer	30–45	
		HRAS	Mutation	Bladder urothelial	57	[32]
		NRAS	Mutation	Melanoma	94	[33, 34]
				Leukemia	59	
RHO	Regulate vesicle transport and assembly and disassembly of actin cytoskeleton required for cell migration and invasion [35].	RHOA	Overexpression	Colon	95	[35]
				Lung	95	
		RAC1	Mutation	Breast	50	[14, 33, 36–39]
			Overexpression	Breast	70	
				Lung	50	
		CDC42	Overexpression	Breast	95	[14, 33, 40]
				Colorectal	60	
ARF	Regulate different steps in intracellular membrane transport [7].	ARF1-ARF6	Unknown	–		[7]
RAB	Regulate vesicular membrane trafficking events such as early endosomal membrane tracking, fusion, and sorting [41].	RAB5	Unknown	–		[8, 41, 42]
RAN	Facilitates transport into and out of the nucleus [9].	RAN	Unknown	–		[9]

Over the last decades, several small GTPases were found to be involved in the development of human carcinomas; hence they have become an interesting subject in cancer research [11, 12]. RAS superfamily of G-proteins genes is the most frequently mutated in cancers accounting for up to 30% of human tumors [13], with KRAS being the most rampantly mutated, accounting for up to 86% of RAS mutations in cancer [13]. Other monomeric G-proteins such as RHOA, CDC42, and RAC1 contribute to cancer progression and are found to be mutated or overexpressed in some tumors [11, 14–17]. Summarized in Table 1 are the incidence rates of some of the aberrant G-proteins in various cancers. Moreover, these proteins are involved in the organization and assembly of the F-actin cytoskeleton and thus regulate the cellular processes of migration and invasion that are commonly dysregulated during cancer development and progression to drive metastasis [18–21].

**Figure 1:**
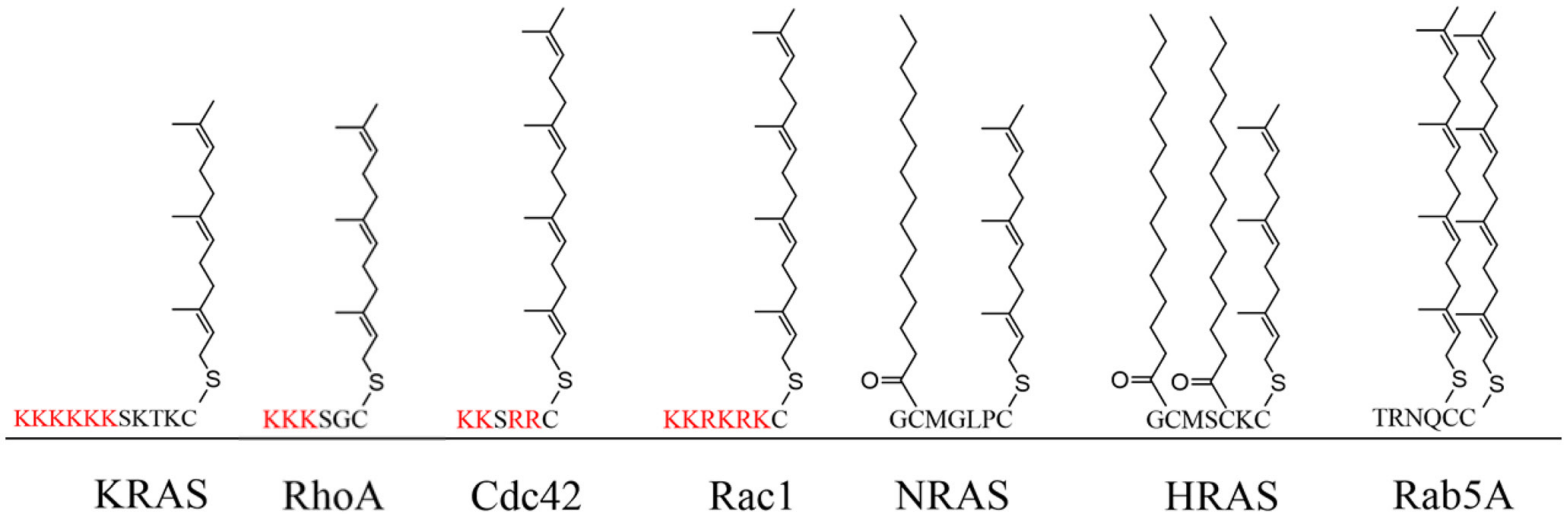
The HVR of the G-proteins and the corresponding PTMs. KRAS and RHOA are farnesylated, RAC1 and CDC42 are geranylgeranylated NRAS and HRAS are both farnesylated and palmitoylated, and Rab5A is doubly geranylgeranylated.

For most G-proteins, their proper functioning is strongly dependent on post-translational modifications (PTMs) [2, 22]. They differ in PTMs, which are characterized by their C-terminal sequence motifs known as hypervariable region (HVR) [23]. The RAS, RHO, and RAB family members are generally C-terminally modified by polyisoprenylation, palmitoylation, or myristoylation [24–26]. However, the ARF/SAR family is mostly modified at their N-terminus by myristoylation, while the Ran family is not modified at all (Table 2) (Figure 1) [2, 27].

**Table 2:** The hypervariable region (HVR) sequence and PTMs of the G-proteins in this study

Sub family	G-protein	HVR sequence	Modified cysteine	PTMs	Cluster of adjacent K and R	References
RAS	KRAS4b	166H**K**E**K**MS**K**DG**KKKKKK**S**K**T**K** CVIM188	C^185^	F	yes	[24]
	KRAS4a	166HKLRKLNPPDESGPGCMSCKCVLS189	C^186^ C^180^	F & P	–	[24]
	HRAS	166H**K**L**RK**LNPPDESGPGCMSC **K** CVLS189	C^181^ C^184^ C^186^	F & P	no	[24]
	NRAS	166Y**R**M**KK**LNSSDDGTQGCMGLPCVVM189	C^181^ C^186^	F & P	no	[24]
Rho	RHOA	171FEMATRAALQA**RR**G**KKK**SGCLVL193	C^190^	F or GG	yes	[43, 44]
	RAC1	171EAIRAVLCPPPV**KKRKRK** CLLL192	C^189^	GG	yes	[44]
	CDC42	171EAILAALEPPEP**KK**S**RR** CVLL191	C^188^	GG	yes	[44]
Rab	RAB5A	191ANSA**R**GRGVDLTEPTQPT**R**NQCCSN215	C^212^ C^213^	2 GG	no	[45]

Abbreviations: F: farnesylation; P: palmitoylation; GG: geranylgeranylation.

The PTMs play numerous roles, such as allowing proper folding, membrane binding and localization, protein-protein interactions, and signaling [46, 47]. There are various protein-protein interactions that involve the polyisoprenyl moiety. For example, KRAS trafficking between various subcellular compartments is facilitated by interactions with several chaperone proteins such as phosphodiesterase δ (PDE-δ), galectins, calmodulin, tubulin, and prenylated RAB-acceptor protein 1 (PRA1) [48]. It has been reported that RAS function can be inhibited using S-farnesyl derivatives of rigid carboxylic acids such as S-*trans,trans* farnesyl thiosalicylic acid (FTS) [49, 50]. The mechanism of action of FTS involves the displacement of RAS from the plasma membrane, thereby accelerating its degradation [51]. It was suspected that the polyisoprenylated cysteinyl amide inhibitors (PCAIs) may function by displacing polyisoprenylated G-proteins from their associations with other macromolecules and cellular structures as membranes. To begin understanding the roles that the PCAIs may play in G-protein function, we determined the effect of PCAIs on two types of monomeric G-proteins, those that are modified with a single polyisoprenyl group (KRAS, RHOA, CDC42 and RAC1) and those that are either doubly polyisoprenylated (RAB5A) or polyisoprenylated and acylated (HRAS and NRAS). Moreover, we also report on the effects of PCAIs on cell migration and invasion, which is controlled by some of these monomeric G-proteins, as well as the PCAIs effect on F-actin cross-linking proteins, vinculin and fascin.

## RESULTS

### PCAIs suppress the viability of the cancer cell lines in a concentration- and polyisoprenylation-dependent manner

**Figure 2:**
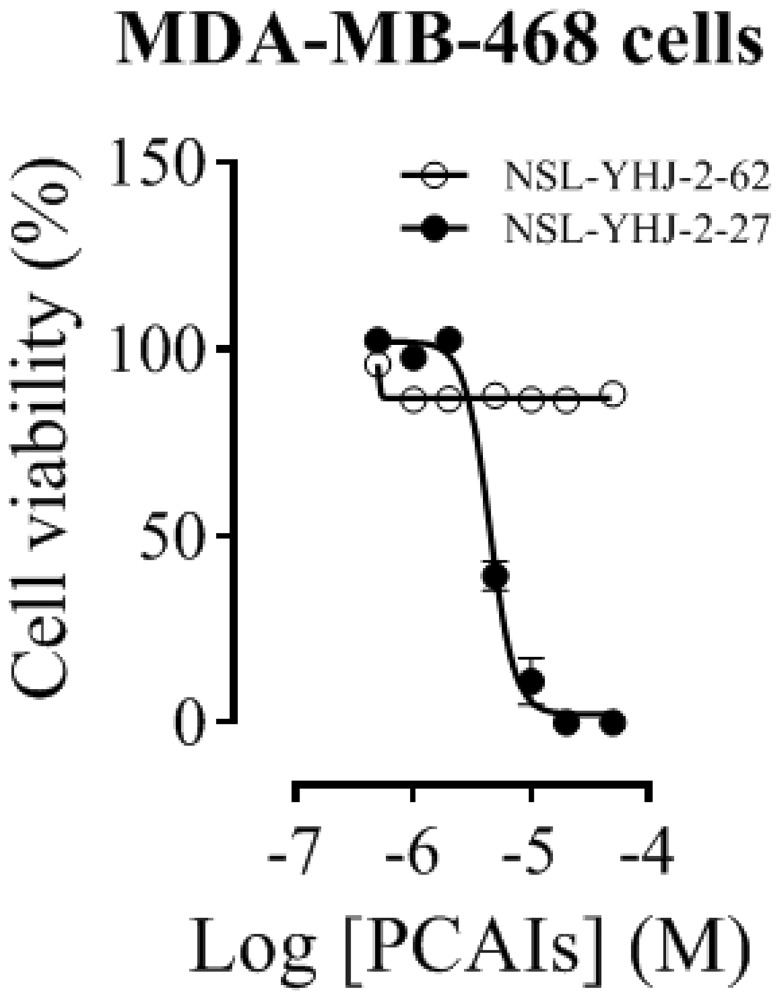
Concentration-response curves of PCAIs against MDA-MB-468 cells. Cells were treated with varying concentrations of NSL-YHJ-2-27 (potent compound) or NSL-YHJ-2-62 (compound lacking the polyisoprenyl moiety used as control) at the onset and after 24 h. After 48 h, resazurin reduction assay was performed to determine the residual cell viability. The EC50 values were determined by plotting the relative fluorescence intensities (expressed percentages of the control values) against concentration in a non-linear regression curve fit using GraphPad Prism version 8.0 for Windows (San Diego, CA, USA).

A significant feature of NSL-YHJ-2-27 is the presence of farnesyl tail which is also found in most G-proteins. Exposure of MDA-MB-468 cells to NSL-YHJ-2-27 resulted in physical changes in the cells as the concentration of the PCAIs increased. Cell viability results show EC_50_ values of 4.4 μM for NSL-YHJ-2-27 and >50 μM for NSL-YHJ-2-62 (Figure 2). The non-farnesylated compound, NSL-YHJ-2-62, did not elicit any changes to the cells. As shown in Table 3, the results obtained from MDA-MB-468 cells corroborate the results presented in a previous paper [52]. Prominent cell rounding started to become visible in cells that were treated with 5 μM NSL-YHJ-2-27, while higher concentrations completely killed the cells. Nonetheless, there were no changes observed on cells treated with NSL-YHJ-2-62 at all the concentrations used (Supplementary Figure 1).

**Table 3:** EC_50_ values of the PCAIs against various cancer cell lines

Compound	48 h EC_50_ (μM)			
	NCI-H1299	A549	MDA-MB-468	MDA-MB-231
NSL-YHJ-2-27	5.3^*^	2.2^*^	2.2^*^	4.4
NSL-YHJ-2-62	>50^*^	>50^*^	>50^*^	>50

The potency of NSL-YHJ-2-27 is apparent in all cell lines, however, the non-polyisoprenylated compound, NSL-YHJ-2-62 did not show any effect on cell viability. ^*^These values have previously been reported as part of a broader study of structure-activity relationships of the PCAIs [52].

### PCAIs deplete singly polyisoprenylated but not doubly polyisoprenylated or polyisoprenylated and acylated G-protein levels

**Figure 3:**
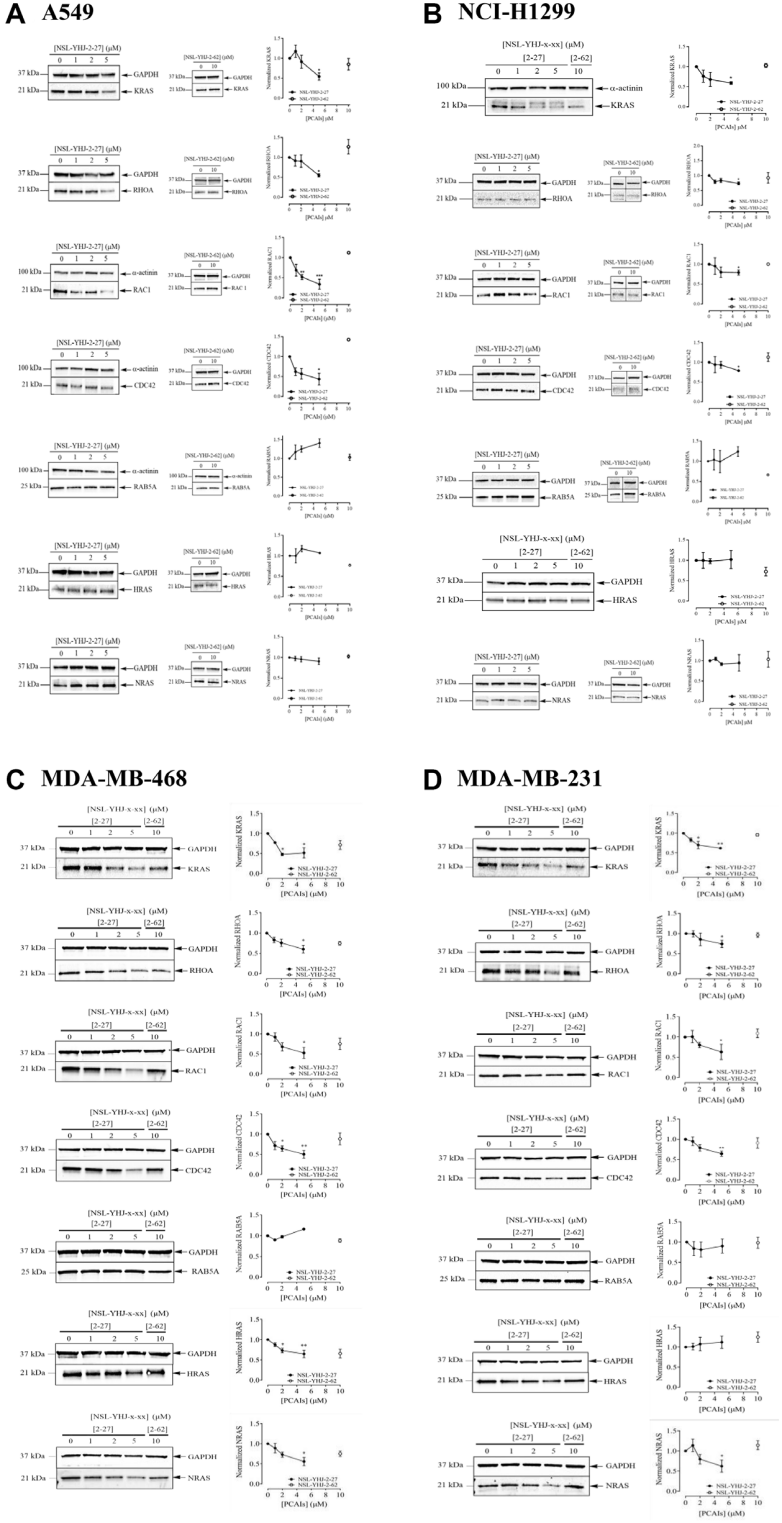
The effect of PCAIs on G-protein levels. Cells were treated for 48 h with 0−5 μM of NSL-YHJ-2-27 (or NSL-YHJ-x-xx where x-xx is 2-27) or 10 μM of its non-farnesylated analog, NSL-YHJ-2-62 (or NSL-YHJ-x-xx where x-xx is 2-62). These were then lysed and subjected to western blot analysis for G-protein levels as described in the methods. (**A**–**D**) Western blot images and densitometry plots of bands following quantification using Image Lab Software were normalized against GAPDH or α-Actinin. The samples were analyzed for G-protein levels of expression in (A) A549 (B) NCI-H1299 (C) MDA-MB-468 and (D) MDA-MB-231 cell lines. Data are representative of three independent experiments. Statistical significance (^*^ *p* < 0.05, ^**^ *p* < 0.01, and ^***^ *p* < 0.001) was determined by 1-way ANOVA with post hoc Dunnett’s test.

To investigate the hypothesized anticancer mechanisms of the PCAIs through disruption of G-protein function, we checked the effects of the PCAIs on the G-protein levels in lung cancer (A549 and NCI-H1299) and breast cancer (MDA-MB-231 and MDA-MB-468) cell lines. When A549 cells were treated with 5 μM of NSL-YHJ-2-27 for 48 h, the KRAS, RHOA, RAC1, and CDC42 protein levels dropped by 46, 45, 57, 66, and 57%, respectively, compared to the control. However, no significant difference was observed in the levels of RAB5A, HRAS, and NRAS (Figure 3A). The same was observed in the NCI-H1299 cells, after treatment with 5 μM of NSL-YHJ-2-27 for 48 h revealed decreased levels of KRAS, RHOA, RAC1, and CDC42 of 40, 27, 20, and 21%, respectively, but no significant changes in RAB5A, HRAS, and NRAS levels (Figure 3B). Furthermore, both breast cancer cell lines exhibited significant reductions in the levels of KRAS, RHOA, RAC1, and CDC42 proteins of 49, 40, 50, and 48%, respectively in MDA-MB-468 cells (Figure 3C). In MDA-MB-231 cells, the levels of the same proteins were reduced by 38, 26, 37, and 36%, respectively (Figure 3D). Contrary to what we initially expected, significant decreases in the levels of HRAS and NRAS by 36 and 44%, respectively were observed in MDA-MB-468 cells (Figure 3C). On the other hand, MDA-MB-231 cells showed a 38% reduction in NRAS protein levels at 5 μM treatment with NSL-YHJ-2-27 (Figure 3D). Of all the proteins tested, the non-farnesylated analog, NSL-YHJ-2-62, did not elicit any significant effects on the cell lines, confirming that the farnesyl moiety is required for the effects of the PCAIs (Figure 3A–3D).

### RAC1 and RHOA are secreted out of the cell after PCAIs treatment

**Figure 4:**
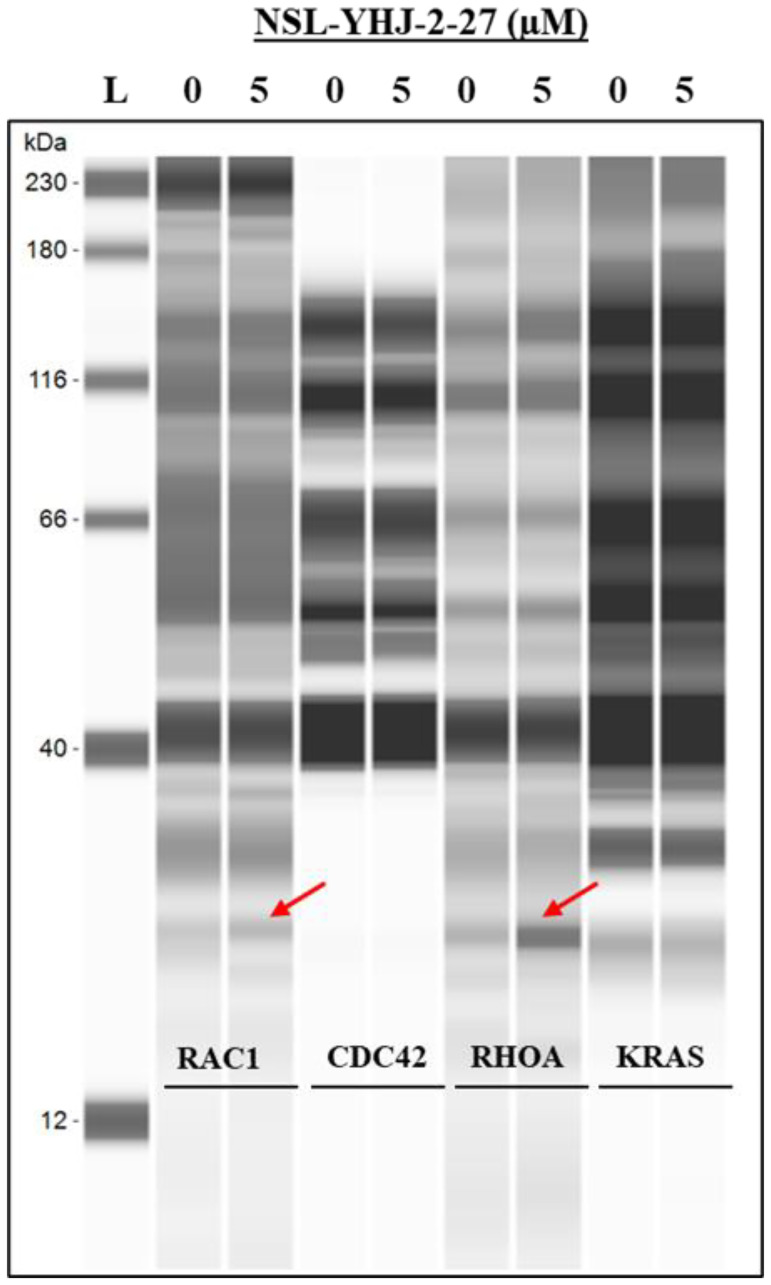
PCAIs induce RAC1 and RHOA secretion from cells. MDA-MB-468 cells were treated with either 0 (control) or 5 μM PCAIs. After 48 h, the media were collected and concentrated using vacuum concentrator (Labconco, USA). The concentrated media were subjected to western blot analysis using Jess Simple western assay, the target proteins (~25 kDA) were probed using the respective antibodies targeting RAC1, CDC42, RHOA, or KRAS.

To understand the mechanism of G-protein depletion following treatment of cells with PCAIs, we set out to determine whether these proteins are degraded and/or secreted from the cell. The results show that RAC1 and RHOA were both secreted into the culture medium, while CDC42 and KRAS were not (Figure 4).

### PCAIs inhibit cancer cell migration and invasion

**Figure 5:**
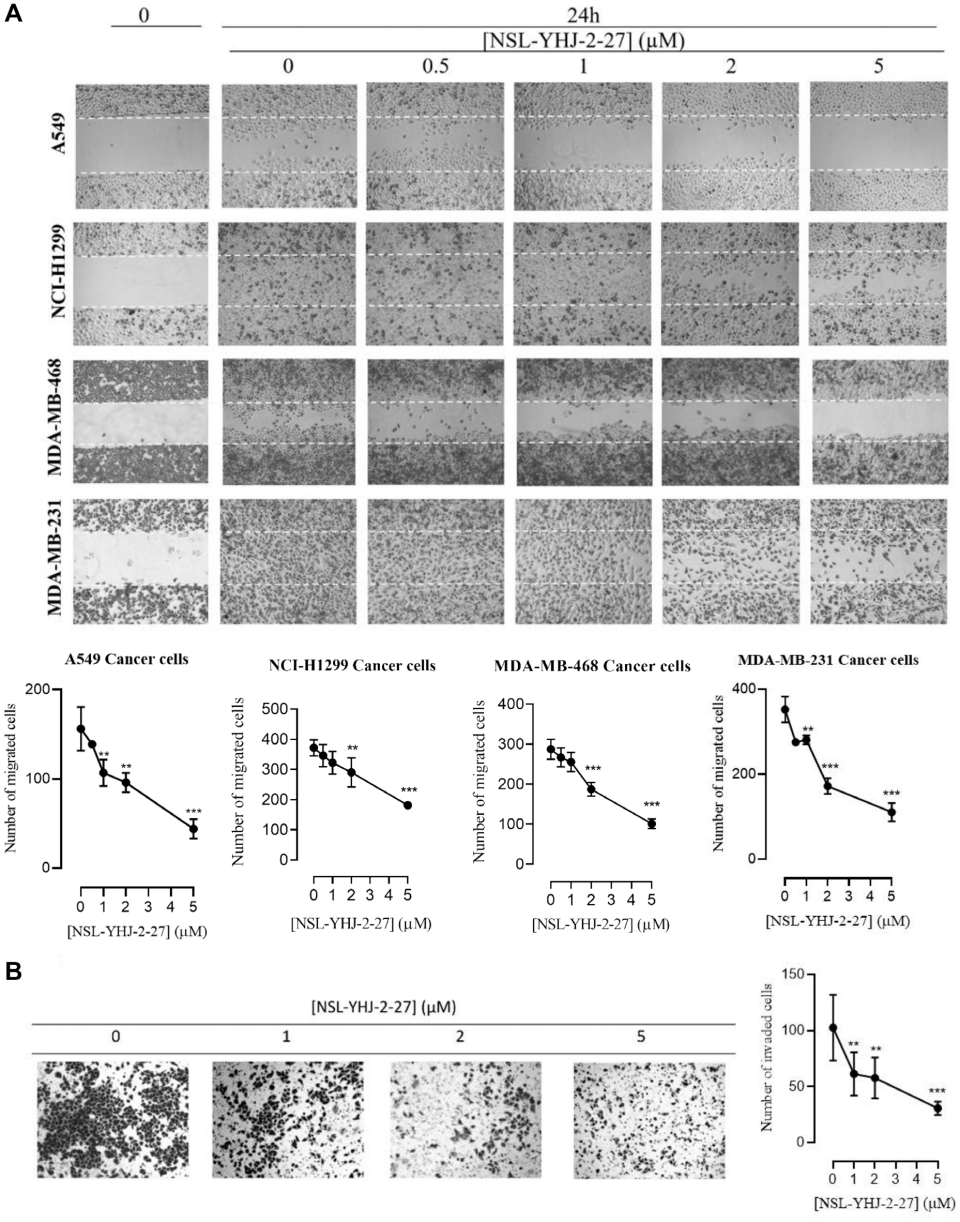
NSL-YHJ-2-27 suppresses cancer cell migration and invasion. (**A**) Confluent monolayers of cancer cells separated by a “wound” generated using cell culture inserts (ibidi) were treated with the indicated concentrations of NSL-YHJ-2-27 and closure of the wounds was monitored, and images captured at 0 and 24 h after treatment using a Nikon Ti Eclipse microscope at 4X magnification. The number of cells that migrated into the wounds were counted. (**B**) A549 cells were plated onto the inserts of 24-well Matrigel invasion chambers after treatment and incubated for 24 h as indicated in the Methods. Cells that invaded from the top chamber of inserts through Matrigel were trapped on the membrane in the lower chamber of the inserts. These invading cells were fixed and then stained with 1% crystal violet. Bright field images were obtained using Nikon Eclipse microscope at 4X magnification. The results are the means of three independents experiments. Statistical significance (^**^ *p* < 0.01, and ^***^ *p* < 0.001) was determined using 1-way ANOVA with post hoc Dunnett’s tests.

For cells to metastasize, they need to migrate from a primary tumor through the extracellular matrix and invade distal tissues. To better understand the potential of the PCAIs at inhibiting metastasis, we tested their effect on cancer cells migration and invasion. Treatment with 5 μM of compound NSL-YHJ-2-27 inhibited the number of migrated cells in A549, NCI-H1299, MDA-MB-468, and MDA-MB-231 cell lines by 72, 41, 46, and 68% respectively, after 24 h as compared to controls (Figure 5A). Furthermore, the effect of PCAIs on cellular invasion was determined using the trans-well invasion assays and the number of cells that were able to invade the extracellular matrix (ECM) and make their way to the other side of the membrane were quantified. Treatment of A549 cells with compound NSL-YHJ-2-27 yielded a significant reduction in the number of cells that invaded the ECM as compared to controls. We observed a concentration-dependent decrease in the number of cells that invaded through Matrigel following exposure to NSL-YHJ-2-27. Exposure to 1, 2, and 5 μM of NSL-YHJ-2-27 suppressed the invasion of A549 cells by 40, 44, and 70% respectively, after 24 h compared to controls (Figure 5B).

### PCAIs decrease the levels of vinculin and fascin in A549 cells

**Figure 6:**
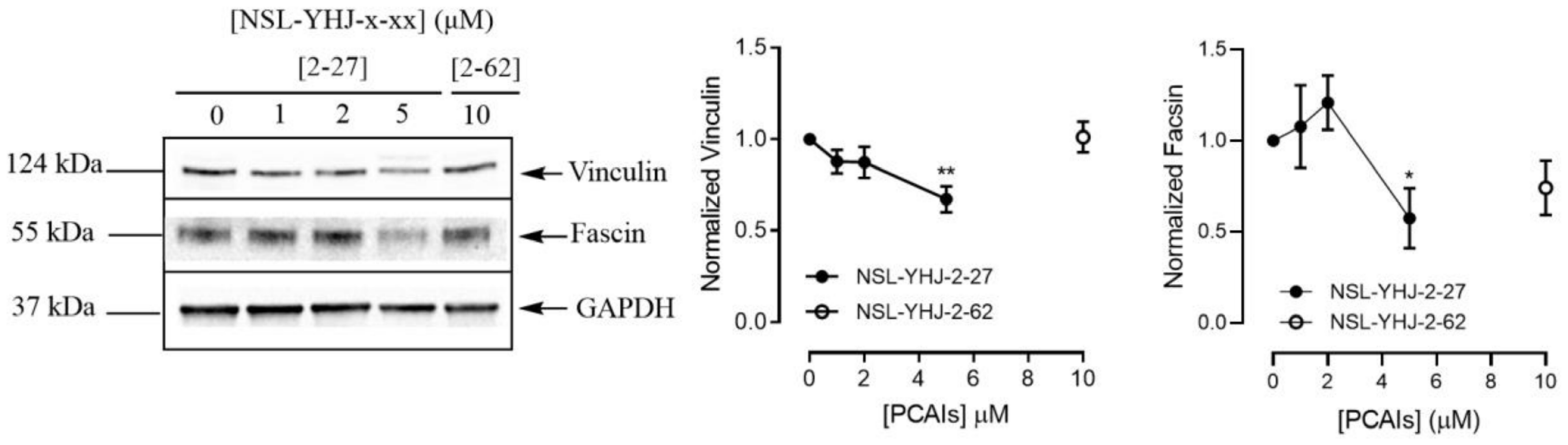
PCAIs decrease the levels of vinculin and fascin proteins. Cells were treated for 48 h with 0−5 μM of NSL-YHJ-2-27 or 10 μM of NSL-YHJ-2-62. These were then lysed and subjected to western blot analysis for vinculin and fascin protein levels as described in the Materials and Methods. Densitometry of bands and quantification were performed using Image Lab Software and normalized to GAPDH. Data are representative of three independent experiments. Statistical significance (^*^ *p* < 0.05 and ^**^ *p* < 0.01) was determined by 1-way ANOVA with post hoc Dunnett’s test.

To understand the effect of PCAIs on cell migration and invasion more, we investigated their effect on the F-actin cross-linking proteins vinculin and fascin that bridge integrins to the actin cytoskeleton [53]. The levels of vinculin protein decreased by 33% and the levels of fascin protein dropped by 43% after exposure of A549 cells to 5 μM of NSL-YHJ-2-27 (Figure 6).

## DISCUSSION

The notion that the PCAIs may directly impact G-protein functions was predicted by their structural similarities to the PTMs on those modified with a single polyisoprenyl moiety and the numerous reports indicating that the farnesylation or geranylgeranylation directly contribute to protein-protein interactions [54–57]. One of the examples of prenylation dependent protein–protein interactions is the interaction with chaperone proteins such as galectin 3, 8, 14-3-3, PRA1, and calmodulin (CALM) in subcellular trafficking [54–57]. For example, galectin 8 isoforms have been shown to bind to farnesylated but not to unfarnesylated KRAS [58]. Moreover, it was found that inhibiting PDEδ with small molecules that bind to the farnesyl-binding pocket of PDEδ can impair KRAS localization to the plasma membrane [59, 60].

While exploring the mechanism of action of FTS, that was reported to inhibit RAS function, it was determined that it releases RAS from the membrane, displacing it into the cytosol, indicating that the major site of action of the FTS is at the anchoring point in the membrane [51]. The PTMs pathway is vital for the functions of most small GTPases where it is essential for their membrane binding and localization which is an essential step in their activation [61]. These modifications facilitate the proteins’ association with the inner surfaces of the plasma membranes, where they interact with upstream activators and downstream effectors in various signaling pathways [57].

Small G-proteins differ in PTMs, which are defined by the varying signal sequences in the HVR [23]. Of the RAS isoforms, KRAS is the only one in which a single C-terminal cysteine is modified by polyisoprenylation [24], while NRAS and HRAS are polyisoprenylated and additionally modified by one or two palmitoyl groups [24]. RHOA, RAC1, and CDC42 also have a single C-terminal polyisoprenylated cysteine, while RAB5 undergoes a double GG modification [44, 45] (Table 2) (Figure 1). These PTM differences may help explain the differences between the effects of the PCAIs on these small G-proteins. The significant suppression of the PCAIs on the levels of KRAS, RHOA, RAC1, and CDC42 that are modified only at one site through polyisoprenylation suggests that the PCAIs are more capable of dislodging them from polyisoprenylation-dependent interactions than they would RAB5A that has an additional modified cysteine. Geranylgeranylation results in higher affinities than farnesylation [62, 63]. This implies that PCAIs competitive displacement of a doubly geranylgeranylated RAB5A would be improbable. The effect of PCAIs on NRAS and HRAS varied depending on cell lines. In A549 and NCI-H1299, the levels of NRAS and HRAS were not affected, while in MDA-MB-468 cells, the said G-proteins decreased, and in MDA-MB-231 cells only NRAS showed a reduction in its protein levels. It isn’t clear how the PCAIs would be able to suppress the NRAS and HRAS levels since the additional acyl modifications that contribute to anchoring the proteins to the membranes would make dislodgement more difficult. That differences in the levels of these G-proteins were only observed in some cell lines is an indication that other unique cellular factors that do not involve direct competitive effects at the protein-protein interaction level may be in play. In fact, palmitoylation is readily reversible under physiologic conditions [64]. The binding and dissociation of RAS proteins modified only through farnesylation from membranes have been reported to occur rapidly than those attached through farnesylation and palmitoylation [25, 26]. RAS isomers that are both farnesylated and palmitoylated have more than 100-fold higher affinity for membranes than only farnesylated RAS [26, 65].

In addition to the PTMs, an accumulation of positively charged amino acids in the polybasic region are also essential for membrane attachments and protein-protein interactions [24, 66] (Table 2) (Figure 1). KRAS, RHOA, RAC1, and CDC42 which all showed decreased levels upon treatment with the PCAIs contain adjacent clusters of basic amino acid residues to bind negatively charged phospholipid headgroups in membranes [24]. The tethered positive charges of the ionized piperizinyl moiety in the PCAIs may somewhat mimic the positive charges of the polybasic regions of G-proteins and may play a similar role when the PCAIs uncouple G-proteins from their polyisoprenyl-dependent interactions.

Overexpression and/or hyper-activation of some members of the RHO family of small GTPases enhance F-actin remodeling, which is central to cell migration and invasion processes involved in metastasis [67]. Therefore, the observed decreases in RhoA, vinculin and fascin levels upon PCAIs treatment explain the significant inhibition of A549 cells migration and invasion given the F-actin cross-linking roles of vinculin and fascin that bridge integrins to the actin cytoskeleton [52]. It has been reported that depletion of vinculin disrupts cell adhesion and promotes apoptosis [52]. Moreover, fascin has been reported to be overexpressed in various cancer types [68]. PCAIs-mediated depletion of vinculin and fascin may be through weakening of integrin-F-actin linkages and enhancing F-actin loss, thereby inhibiting cell migration and invasion. Other PCAIs were shown to disrupt vinculin punctates in NCI-H1299 cells [69, 70].

The proposed mechanism of action on how the PCAIs interact with the respective G-proteins were mainly based on their structure and how they can displace the target proteins. The effects of the PCAIs on the G-proteins may be due to direct physical competitive displacement that may result in more rapid degradation than when they are in complex with other proteins. It is still uncertain what happens to the proteins after they got displaced but based on our results, some of them appear to be degraded and secreted out. We hypothesize that the secretory pathway may be involved in the depletion of RAC1 and RHOA since the PCAIs treatment resulted in significant amounts of both proteins in the experimental media. Other processes such as feedback control on G-proteins may more accurately explain the changes in NRAS and HRAS only in some cells.

Furthermore, our previous results show that PCAIs induce the phosphorylation activation of the MAP kinase pathway enzymes resulting in the phosphorylation of p90RSK [56]. Phosphorylated p90RSK is known to inhibit Son-of-Sevenless (SoS) [71]. p90RSK regulates cAMP-response element binding protein (CREB) [72, 73], thereby affecting gene transcription. It may also alter translation by phosphorylating ribosomal proteins [71]. The latter two effects would alter intracellular protein levels that may include changes in the NRAS and HRAS proteins.

In conclusion, mutations in G-proteins have been associated in the progress of several cancers, thus, a new approach on developing new anticancer therapies by targeting these proteins will be tantamount to finding the cure. Our results show that PCAIs deplete the protein levels of some significant G-proteins which are known to be involved in the migration and invasion of cells (i.e., metastasis) such as RAC1, RHOA, and CDC42. Furthermore, the PCAIs also affect the expression of vinculin and fascin which are both important for cell motility by forming F-actin linkages. The initial findings presented here indicate how PCAIs can be used as potent agents in developing new anticancer therapeutics, therefore, more extensive studies need to be done to elucidate on its potency. Although we cannot conclusively explain the exact mechanism of action of PCAIs on how they affect the levels of some G-proteins yet, but we can say that these PCAIs have the ability to affect the progression of cancer.

## MATERIALS AND METHODS

### Materials

Cell lines were purchased from American Type Culture Collection (ATCC, Manassas, VA, USA). Antibodies specific to KRAS (Cat. #53270), RHOA (Cat. #2117), RAC1/2/3 (Cat. #2465), RAB5A (Cat. #46449), CDC42 (Cat. #2462), Vinculin (Cat. #18799), Fascin (Cat. #54545) GAPDH (HRP Conjugate) (Cat. #8884), α-Actinin (HRP Conjugate) (Cat. #12413), anti-mouse IgG, HRP-linked Antibody (Cat. #7076), and anti-rabbit IgG, HRP-linked Antibody (Cat. #7074) were purchased from Cell Signaling Technology (Danvers, MA, USA). Antibodies specific to HRAS (MAB3617) and NRAS (MAB10009) were purchased from Thermo Scientific (Waltham, MA, USA). The PCAIs used in this study (Table 4) were synthesized in our lab as previously described [74, 75].

**Table 4:** Structures of the PCAIs used in this study

Compound	Structure
NSL-YHJ-2-27	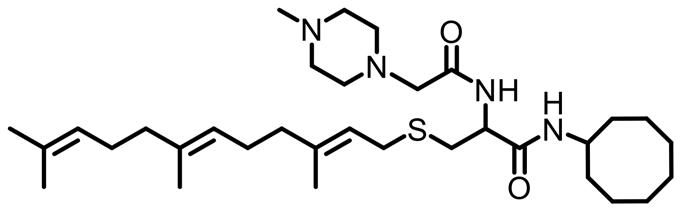
NSL-YHJ-2-62	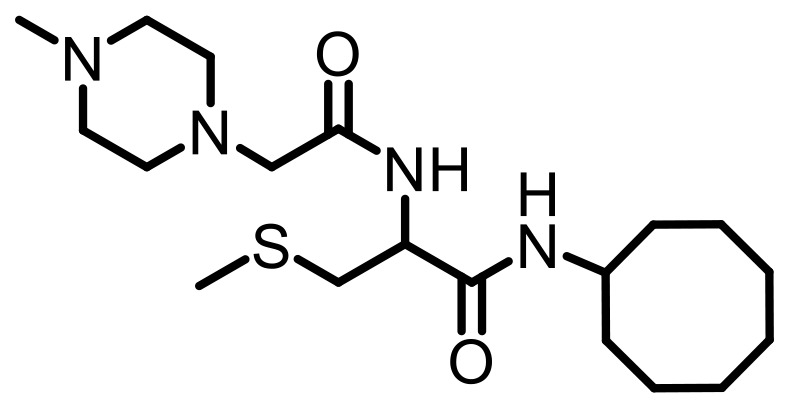

Potent PCAIs analog NSL-YHJ-2-27 and analog lacking the polyisoprenyl moiety NSL-YHJ-2-62 used as control compound.

### Cell culture

A549 (CCL-185)- collected from a 58-year old Caucasian male, it is a hypotriploid human cell line with the modal chromosome number 66 which can be found in 24% of cells, NCI-H1299 (CRL-5803)- established from a lymph node of 43-year old White male patient with lung cancer who received prior radiation therapy, MDA-MB-231(HTB-26)- obtained from a 51-year old White female, it is an aneuploid female with a modal chromosome number 64, and MDA-MB-468 (HTB-132)- isolated from a pleural effusion of a 51-year old Black woman with a metastatic breast adenocarcinoma, an aneuploid female with most chromosome counts in the hypertriploid range with a modal chromosome number 64. The A549, MDA-MB-231, and MDA-MB-468 cells were cultured in high glucose Modified Eagle Medium (DMEM) (Genesee Scientific, San Diego, CA, USA) while NCI-H1299 cells were cultured in RPMI 1640 (Genesee Scientific, San Diego, CA, USA). All media were supplemented with 10% heat-inactivated fetal bovine serum (Genesee Scientific, San Diego, CA, USA), 100 U/mL penicillin and 100 μg/mL streptomycin (Genesee Scientific, San Diego, CA, USA). The cultures were incubated at 37ºC in 5% CO_2_/95% humidified air. In all cases, treatment with experimental compounds was done in basal medium supplemented with 5% heat-inactivated fetal bovine serum.

### Effects of PCAIs on MDA-MB-468 cell line

To determine the potency of the PCAIs, NSL-YHJ-2-27 and the control analog, NSL-YHJ-2-62, cell viability assay was conducted. Briefly, 1 × 10^4^ cells/well of MDA-MB-468 cells were plated into 96-well culture plates (Genesee Scientific, San Diego, CA, USA) in experimental medium. When the cells adhered to the wells, the respective analogs were added to final concentrations of 0.5, 1, 2, 5, 10, 20, 50 μM at the beginning and after 24 h of incubation. Acetone (1% final concentration) used as the vehicle solvent was used for the control treatment. The cells were exposed to the compounds for 48 h after which bright-field microscope images (10× magnification) were captured using the Nikon Eclipse microscope to evaluate physical changes on the cells. Then resazurin reduction assay was conducted by adding 0.02% of resazurin reagent dissolved in PBS into the cells. They were then incubated at 37ºC in 5% CO_2_/95% humidified air for 2 h. Using SoftMax Pro Reader version 5.4 for Windows (Molecular Devices, CA, USA), the fluorescence intensities were determined by setting the excitation frequency at 544 nm and emission at 590 nm. The cell viability was expressed as the percentage of the fluorescence in the cells treated with the compounds relative to the control (0 μM). These were then plotted in a non-linear regression curve fit using GraphPad Prism version 8.0 for Windows (San Diego, CA, USA) to determine the EC_50_ value for each compound.

### Effect of PCAIs on the G-proteins

Cells in complete medium were plated into 60.8 cm^2^ tissue culture dishes (Genesee Scientific, San Diego, CA, USA) at a cell density of 7 × 10^5^ (or 1 × 10^6^) cells/dish and then incubated for 24 h to allow the cells to adhere. Adherent cells were treated with varying concentrations of PCAIs (0–5 μM) in experimental medium (supplemented with 5% heat-inactivated FBS). After 24 h, equivalent amounts of PCAIs were used to treat the cells for the 48 h exposure. Cells were washed with PBS and then lysed with RIPA buffer (Genesee Scientific, San Diego, CA, USA) supplemented with 0.1% v/v protease/phosphatase inhibitor cocktail (Cell Signaling Technology, Danvers, MA, USA). The amount of protein in lysates was determined using the Quick Start™ Bradford protein assay (Bio-Rad, Hercules, CA, USA). Cell lysates containing equal protein amounts (20–30 μg) were boiled in XT sample buffer with XT reducing agent (Bio-Rad, Hercules, CA, USA). The samples were separated by SDS-PAGE on 12% Criterion™ XT Bis-Tris protein gels and transferred onto Trans-Blot turbo midi 0.2 μm nitrocellulose membranes (Bio-Rad, Hercules, CA, USA). Membranes were blocked for 1 h at room temperature with OneBlock™ western-CL blocking buffer (Genesee Scientific, San Diego, CA, USA) and incubated overnight in blocking buffer containing respective monoclonal antibodies against the target proteins at 4°C. Membranes were then washed with 1X TBST and incubated with anti-rabbit or anti-mouse IgG, HRP-linked antibodies at room temperature for 2 h. Immunoreactive bands were then visualized using ProSignal^®^ Pico (Genesee Scientific, San Diego, CA, USA) or Radiance Plus (Azure Biosystems, Dublin, CA, USA) ECL reagents per manufacturer’s recommendations using the ChemiDoc XRS+ System (Bio-Rad, Hercules CA, USA). Protein levels as judged by the chemiluminescent intensities were quantified using Image Lab 6.0 (Bio-Rad, Hercules CA, USA), normalized against the corresponding band intensities of either GAPDH or α-Actinin. The results from three independent trials were then plotted using GraphPad Prism version 8.0 for Windows (San Diego, CA, USA).

### Effect of PCAIs on the degradation of G-proteins

MDA-MB-468 cells were plated into 60.8 cm^2^ tissue culture dishes (Genesee Scientific, San Diego, CA, USA) at a cell density of 1.5 × 10^6^ cells/dish in complete medium and then incubated for 24 h to allow the cells to adhere. Adherent cells were treated with 0 (control) or 5 μM concentrations of PCAIs in experimental medium. After 24 h, PCAIs treatments were repeated followed by a further 24 h incubation. The presence of G-proteins in the incubation media was then determined as follows. Using a vacuum concentrator (Labconco, USA), 1.5 mL of collected media mixed with 4× loading buffer and 20× reducing agent were vacuum concentrated to 200 μL over 1 h at 30°C. The concentrated media were subjected to Western blotting using respective antibodies to detect the target proteins, RAC1, CDC42, RHOA, and KRAS. Simple Western Blotting using Jess assay (Protein Simple, Bio-Techne, MN, USA) was used to analyze the results. Experiments were done according to the manufacturer’s protocol. Briefly, the concentrated media were mixed with 0.1× sample buffer and 5× master mix in 600 μL tube. The samples were denatured for 5 minutes at 95°C and were kept on ice during loading. Running conditions were set in the machine – 30 min blocking, 1 h primary antibody and 1 h secondary antibody.

### Effect of PCAIs on cell migration

The effects of PCAIs on cellular migration were determined using the wound healing assay. Cell culture inserts purchased from ibidi (Martinsried, GE) were used according to the manufacturer’s protocol to generate two confluent monolayers of cells separated by a “wound” for this assay as previously described [70]. Briefly, cells (2–4 × 10^5^ cells/mL) were seeded into each side of the ibidi-cell culture inserts attached onto the wells of a 12-well plate. The plate was then incubated (37°C/5% CO_2_) overnight to allow the cells to attach onto the plate and form two adherent confluent monolayers of cells on either side of the tissue culture insert. The next day, the insert was gently removed to generate a gap or “wound” between the two confluent layers of cells. The monolayers were washed with experimental media once and then fresh experimental media containing varying concentrations of NSL-YHJ-2-27 (0–5 μM) were added. Bright-field microscope images around the “wound” were captured at 0 and 24 h using the Nikon Eclipse microscope. The number of cells that migrated into the “wounds” were counted for control and treated cells. Data were analyzed using GraphPad Prism version 8.0 for Windows (San Diego, CA, USA).

### Effect of PCAIs on cell invasion

The effects of PCAIs on cellular invasion were determined using the transwell invasion assay. The 24-well BD Biocoat Matrigel invasion chambers and inserts (catalog #354480) (Corning, Bedford, MA, USA) were used according to the manufacturer’s protocol. Briefly, A549 cells were harvested and plated in T-25 flasks at 2.0 × 10^5^ cells per flask and allowed to attach overnight. The following day media was replaced with media containing NSL-YHJ-2-27 (0–5 μM) and incubated for 24 h. The 24-well BD Biocoat Matrigel invasion chambers and inserts were rehydrated per manufacturer’s protocol with serum free media and incubated at 37°C for 2 h. The treated cells were harvested to create a cell suspension of 5.0 × 10^4^ cells/mL. Rehydrating media was removed from the wells and inserts. Media containing 10% FBS was added to the wells while 500 μL of the cell suspension was added into the inserts. Invasion inserts containing cell suspensions were then carefully transferred into the wells containing media with 10% FBS. The cells were incubated for 24 h (37 C/5% CO_2_) to allow the cells to invade from the upper chamber through the Matrigel into the lower chambers of the inserts. After incubation, non-invasive cells were quickly removed with a cotton swab. Invasive cells remaining in the inserts were fixed with 4% formaldehyde in PBS for 20 min and then stained with 1% crystal violet. Invading cells were imaged using Nikon Eclipse microscope, quantified using the Nikon NIS-Elements software, analyzed using GraphPad Prism, and plotted as the means numbers of migrated cells against NSL-YHJ-2-27 concentration.

### Effect of PCAIs on vinculin and fascin

Cells in complete medium were plated into 60.8 cm^2^ tissue culture dishes (Genesee Scientific, San Diego, CA, USA) at a cell density of 7 × 10^5^ cells/dish and then incubated for 24 h to allow the cells to adhere. Adherent cells were treated with 0–5 μM of PCAIs in experimental medium. After 24 h, equivalent amounts of PCAIs were used to treat the cells for the 48 h exposure. Cells were washed with PBS and then lysed with RIPA buffer (Genesee Scientific, San Diego, CA, USA) supplemented with 0.1% v/v protease/phosphatase inhibitor cocktail (Cell Signaling Technology, Danvers, MA, USA). The amount of protein in lysates was determined using the Quick Start™ Bradford protein assay (Bio-Rad, Hercules, CA, USA). Cell lysates containing equal protein amounts (20–30 μg) were boiled in XT sample buffer with XT reducing agent (Bio-Rad, Hercules, CA, USA). The samples were separated by SDS-PAGE on a 4%–12% Criterion™ XT Bis-Tris protein gels and transferred onto Trans-Blot turbo midi 0.2 μm nitrocellulose membranes (Bio-Rad, Hercules, CA, USA). Membranes were blocked for 1 h at room temperature with OneBlock™ western-CL blocking buffer (Genesee Scientific, San Diego, CA, USA) and incubated overnight in blocking buffer containing monoclonal antibodies against the target proteins vinculin and fascin at 4°C. Membranes were then washed with 1X TBST and incubated with anti-rabbit or anti-mouse IgG, HRP-linked antibodies at room temperature for 2 h. Immunoreactive bands were then visualized using ProSignal^®^ Pico (Genesee Scientific, San Diego, CA, USA) or Radiance Plus (Azure Biosystems, Dublin, CA, USA) ECL reagents per manufacturer’s recommendations using the ChemiDoc XRS+ System (Bio-Rad, Hercules CA, USA). Protein levels as judged by the chemiluminescent intensities were quantified using Image Lab 6.0 (Bio-Rad, Hercules CA, USA), normalized against the corresponding band intensities of either GAPDH or α-Actinin. The results were then plotted using GraphPad Prism version 8.0 for Windows (San Diego, CA, USA).

### Statistical analysis

All results are the means ± SEM. To determine statistical significance, the values of each treatment group were compared to the respective controls by One-Way ANOVA with Dunnett’s post-hoc test using GraphPad Prism version 8.0 for Windows (San Diego, CA, USA) and *p* values less than 0.05 were considered significant.

## SUPPLEMENTARY MATERIALS


