# Supplementary material for: Polyisoprenylated cysteinyl amide inhibitors deplete singly polyisoprenylated monomeric G-proteins in lung and breast cancer cell lines
Source: Oncotarget. 2023 Mar 24;14:243–57. doi: 10.18632/oncotarget.28390 (PMC10038354; doi:10.18632/oncotarget.28390)
Supplement: Supplementary file 1 [file oncotarget-14-28390-s001.pdf]

# Polyisoprenylated cysteinyl amide inhibitors deplete singly polyisoprenylated monomeric G-proteins in lung and breast cancer cell lines

## SUPPLEMENTARY MATERIALS

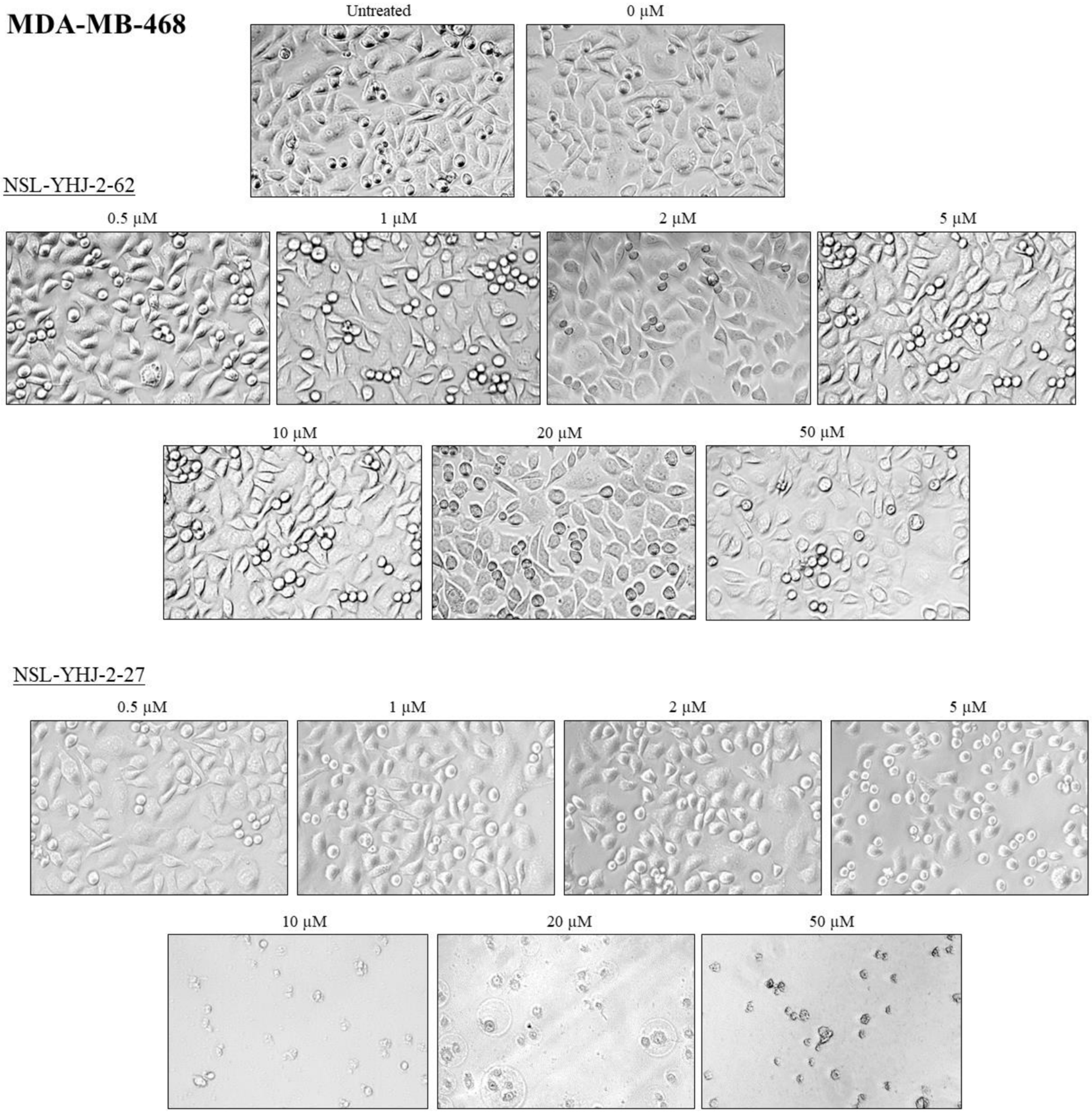


**Supplementary Figure 1: PCAIs affect cell viability and cause physical changes in MDA-MB-468 cells in a dose-dependent manner.** Cells at a density of $1 \times 10^4$ cells/well were plated into 96-well culture plate in experimental medium. The cells were allowed to adhere to the well overnight then respective concentrations of PCAIs (0.5, 1, 2, 5, 10, 20, 50 µM) were treated into the cells in four replicates for each concentration. Bright field images were obtained using Nikon Eclipse microscope at 10x magnification.
